# The effect of maternal pregestational diabetes on fetal autonomic nervous system

**DOI:** 10.14814/phy2.15680

**Published:** 2023-05-05

**Authors:** Luis Mercado, Diana Escalona‐Vargas, Sarah Blossom, Eric R. Siegel, Julie R. Whittington, Hubert Preissl, Kaitlyn Walden, Hari Eswaran

**Affiliations:** ^1^ Division of Maternal‐Fetal Medicine, Department of Obstetrics and Gynecology University of Arkansas for Medical Sciences Little Rock Arkansas USA; ^2^ Department of Pediatrics University of Arkansas for Medical Sciences Arkansas Children's Research Institute Little Rock Arkansas USA; ^3^ Department of Biostatistics University of Arkansas for Medical Sciences Little Rock Arkansas USA; ^4^ Institute for Diabetes Research and Metabolic Diseases of the Helmholtz Center Munich at the University of Tübingen German Center for Diabetes Research (DZD) Tübingen Germany

**Keywords:** autonomic nervous system, fetus, heart rate variability, magnetocardiography, neurodevelopment, pregestational diabetes, pregnancy, Type 1, Type 2

## Abstract

Heart rate variability assessment of neonates of pregestational diabetic mothers have shown alterations in the autonomic nervous system (ANS). The objective was to study the effect of maternal pregestational diabetes on ANS at the fetal stage by combining cardiac and movement parameters using a non‐invasive fetal magnetocardiography (fMCG) technique. This is an observational study with 40 participants where fetuses from a group of 9 Type 1, 19 Type 2 diabetic, and 12 non‐diabetic pregnant women were included. Time and frequency domain fetal heart rate variability (fHRV) and coupling of movement and heart rate acceleration parameters related to fetal ANS were analyzed. Group differences were investigated using analysis of covariance to adjust for gestational age (GA). When compared to non‐diabetics, the Type 1 diabetics had a 65% increase in average ratio of very low‐frequency (VLF) to low‐frequency (LF) bands and 63% average decrease in coupling index after adjusting for GA. Comparing Type 2 diabetics to non‐diabetics, there was an average decrease in the VLF (50%) and LF bands (63%). Diabetics with poor glycemic control had a higher average VLF/LF (49%) than diabetics with good glycemic control. No significant changes at *p* < 0.05 were observed in high‐frequency (HF) frequency domain parameters or their ratios, or in the time domain. Fetuses of pregestational diabetic mothers exhibited some differences in fHRV frequency domain and heart rate‐movement coupling when compared to non‐diabetics but the effect of fHRV related to fetal ANS and sympathovagal balance were not as conclusive as observed in the neonates of pregestational diabetic mothers.

## Graphical abstract

Fetuses of pregestational diabetic mothers exhibited differences in heart rate variability in frequency‐domain related to sympathovagal balance in comparison to controls. Fetuses of maternal diabetics with poor glycemic control compared to good glycemic control indicated a shift toward sympathetic activity. Fetal ANS can potentially be altered by maternal pregestational diabetes, especially with regard to fetal heart rate variability. Observations are consistent with neonates of pregestational diabetic mothers, thus, indicating the origin of the effect occurring in the prenatal period.

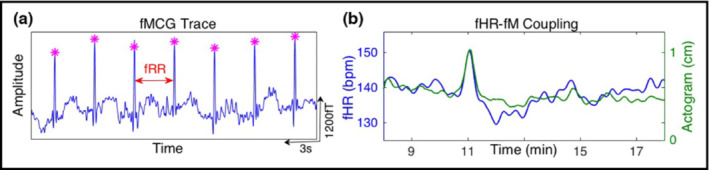

## 1 INTRODUCTION

The prevalence of Type 1 and Type 2 diabetes represents a health concern for women of childbearing age. Over a 15‐year period, the prevalence of births complicated by pregestational diabetes increased by 44% in Type 1 diabetes and 90% in Type 2 diabetes (Mackin et al., 2018). Pregestational diabetes is one of the most common risk factors for maternal, fetal, and infant complications (Wilmot & Mansell, 2014). While improvements in maternal diabetes care have decreased the incidence of adverse neonatal and infant outcomes, risks to infant's health remain elevated in pre‐diabetic pregnancy when compared with the general population (Boinpally & Jovanovič, 2009; Evers et al., 2004; Murphy et al., 2008). Maternal medical comorbidities, like hypertension and obesity, along with poor glycemic control are likely etiological factors (Boinpally & Jovanovič, 2009; Evers et al., 2004; Murphy et al., 2008).

Despite diligent attention to blood sugar monitoring, the risk of adverse maternal and infant outcomes is higher in diabetic pregnancy compared to non‐diabetic pregnancies (Nold & Georgieff, 2004). In general, it is recommended to have a prepregnancy target of glycated hemoglobin (HbA_1c_) level of <6%. A recent retrospective cohort study on pregestational diabetic mothers with an HbA_1c_ target of <6.5% at >26 weeks reduced rates of obstetric and neonatal adverse outcomes irrespective of the early values (Finneran et al., 2020). It has been documented that neonates of mothers with Type 1 diabetes show alterations in the autonomic nervous system (ANS) assessed by heart rate variability (Russell et al., 2016). Independent of mode of delivery, researchers found that infants of diabetic mothers had altered cardiac structure and function along with disruption of the ANS (Russell et al., 2008, 2016). Further, a recent study showed that gestational diabetes alters the fetal heart rate's variability during an oral glucose tolerance test (Fehlert et al., 2017).

Studies have shown that non‐invasive biomagnetic recordings provide the opportunity to assess fetal cardiac (fMCG—fetal magnetocardiography) electrophysiology (Gustafson et al., 2012; Lowery et al., 2008). These MCG tracings provide cardiac parameters, and are a biomagnetic analogue of electrocardiography. In this study, we explored the use of fMCG to assess the effect of maternal pregestational diabetes on the fetal ANS by combining fetal cardiac and movement parameters.

## 2 MATERIALS AND METHODS

### 2.1 Data collection

Biomagnetic signals were recorded using a SQUID‐based non‐invasive 151‐sensor SARA (SQUID Array for Reproductive Assessment) system (Lowery et al., 2008). The recordings for the diabetic subjects were collected at bandwidth of 0–100 Hz with a sampling rate of 312.5 Hz and lasted for about 20 min (Mean: 21 min; SD: ±6 min). As described in Avci et al. (2020), 28 pregnant diabetic mothers (9 Type 1 and 19 Type 2) participated in this study. Although the women were encouraged to participate in three recordings during the third trimester, only six of them completed all three recordings. Overall, the participants completed one to three biomagnetic recordings in the range of 28–39 weeks of gestational age (GA) providing 58 recordings in total. In order to assure independence of data points for subsequent analysis we selected only one (i.e., last) recording per subject. Further, since there are 12 weeks in the range of 28–39 GA, each week was filled in with one recording from our existing low‐risk pregnancy database to create a reference group. In total, data from 40 participants were subjected to data extraction and analysis. All participants provided informed written consent and the study was approved by the University of Arkansas for Medical Sciences Institutional Review Board (Protocol Numbers #04234 on June 14th, 2017 and #206700 on June 5th, 2017).

The raw SARA recording consists of a complex mixture of data including maternal heart, fetal heart, movements, maternal and fetal breathing. To analyze the fetal heart data, that is, fetal MCG, the interfering maternal heart‐signal components were removed by applying a spatial‐filter‐based orthogonal projection algorithm (Vrba et al., 2004). Using a threshold detection technique, the beat‐to‐beat intervals were allocated on all fMCG signals, which were used to calculate the fetal heart rate variability (fHRV). Manual correction was implemented for any abnormal beats, false negatives, or false positive detections. To perform a frequency analysis of the fHRV, a linear interpolation at 10 Hz was carried out to resample the beat‐to‐beat intervals, thus providing a uniformly sampled fHR signal (Gustafson et al., 2011, 2012).

### 2.2 Measures

#### 2.2.1 Fetal cardiac activity

FHRV was quantified using the following standard time domain measures: root mean square of successive differences (RMSSD), standard deviation of normal‐to‐normal beat (SDNN), approximate entropy (ApEn), and the fraction of normal‐to‐normal intervals that differ by more than 10 (pNN10), 15 (pNN15), and 20 (pNN20) milliseconds from the previous normal‐to‐normal interval (Camm, 1996). A quantitative geometrical analysis of RR‐intervals was illustrated with a Poincaré plot, which is a scatterplot of each RR‐interval against the next RR‐interval. The ellipse‐fitting technique was used to characterize the Poincaré plot by obtaining the SDs (standard deviations) along the minor (SD1) and major (SD2) axes of the best‐fitting ellipse, which represent the short‐ and long‐term variability, respectively (Karmakar et al., 2009).

In the frequency domain, nonparametric power spectral density estimation was carried out using the Welch method. Since the fMCG recordings were of varying length, in order to maintain consistency first 10 min of data was used for power spectral density calculation. The RR intervals were divided into 20‐s epochs with an overlap of 50% between adjacent epochs. This power spectral density was then integrated within the different frequency bands as follows: very low frequency (VLF, 0.02–0.08 Hz), low frequency (LF, 0.08–0.2 Hz), intermediate frequency (Int, 0.2–0.4 Hz), and high frequency (HF, 0.4–1.7 Hz) (David et al., 2007). The resulting integrals then were transformed to their natural logarithms. We based our frequency bands on previous fetal MCG studies (Gustafson et al., 2011, 2012; Gustafson & Popescu, 2016) including the one by David et al. (2007) on normative fetal ECG spectral analysis. The rationale of their proposed bands was based on the fact that they observed that most of the power was concentrated in VLF and LF bands and there was not much power or “gap” in the Int band. The range of these spectral bands are different from adult HRV; however, the exact ranges useful for fetal monitoring remain unknown and in disagreement in the literature (Romano et al., 2016; van Laar et al., 2008). Based on the band nomenclature described, the following power ratios (or quotients) were calculated for each fetus: VLF/LF, VLF/HF, and LF/HF.

#### 2.2.2 Fetal movement and heart rate coupling

Fetal movement (fM) was identified and quantified through a computational technique called actography (Govindan et al., 2011), which is derived from fMCG peak amplitude and its signal distribution over SARA channels. The fMCG‐based actography reveals any spatial change in fetal location or orientation, and thus, enables us to monitor movement periods within the recording. In addition to identifying fMs, we also extracted the periods of acceleration in fetal heart rate (fHR). As previously reported in Baser et al. (1992) and DiPietro et al. (1996), fHR‐fM coupling was defined as the occurrence of an fM accompanied by an fHR that increased by ≥5 bpm over baseline within 5 s before the start or within 15 s after the start of the fM. The coupling index was calculated as the proportion of the number of coupled fMs to the number of all identified fMs. If coupling was detected, the absolute values of latency between the onsets of the fHR and fM were obtained and duration of coupled fM was measured. Only the recordings with at least one coupled fM were included in the group comparisons of latency and duration, since these two coupling metrics cannot be computed when there are no coupled segments.

### 2.3 Statistical analysis

First, we analyzed maternal and fetal characteristics among the Type 1, Type 2, and Reference groups. Continuous characteristics were summarized using group means and SDs, and assessed for differences among groups using the Kruskal–Wallis test. Categorical variables were summarized using the frequency and proportion, and assessed for group differences with the chi‐square test. Numeric *p*‐values are reported for assessing differences in maternal and fetal characteristics, and are evaluated for significance at 5% alpha. Next, we assessed the effect of diabetes etiology on measures of fHRV and fHR‐fM coupling. Each measure was summarized by group as means and SDs, then compared pairwise for group differences while adjusting for confounding with GA using an ANCOVA approach. Specifically, we used ANCOVA with *Group* as the factor of interest and GA as the continuous covariate to analyze all HRV measures and the continuous measures of HR‐movement coupling. We used negative binomial regression with *Group* as the factor of interest and GA as the continuous covariate to analyze the following three count‐based measures of fHR‐fM coupling: number per minute of fMs, number per minute of coupled fMs, and coupling index.

To investigate the effect of maternal glucose control, we excluded the non‐diabetic participants and classified the diabetic participants as having good control if their glycated hemoglobin (HbA_1c_) level was <6%, or as having poor control if their HbA_1c_ level was ≥6%. The resulting glucose‐control groups were compared for differences while adjusting for GA using ANCOVA for all continuous measures and negative binomial regression for the three count‐based measures. Analysis results for fHRV and fHR‐fM coupling are reported as means (standard deviations [SDs]) and as differences (95% confidence intervals [CIs]). All means (SDs) are unadjusted group means (SDs), that is, from before GA‐adjustment. All ANCOVA‐based differences (95% CIs) are GA‐adjusted differences (95% CIs). However, differences (95% CIs) in fM, coupled fM, and coupling index from the negative‐binomial regressions are GA‐adjusted differences (95% CIs) in the natural logs of count rates. For easier interpretation of GA‐adjusted differences in natural logs, we exponentiated those log‐scale differences and expressed the resulting ratios in the text as percentage‐unit differences from 100%. Numeric *p*‐values for the GA‐adjusted differences in fHRV and coupling are not reported, but differences whose 95% CIs exclude the value 0 are noted as statistically significant at 5% alpha. Adjustment for multiple comparisons was not performed in order to not inflate Type II error in this modestly powered exploratory study.

## 3 RESULTS

**FIGURE 1:**
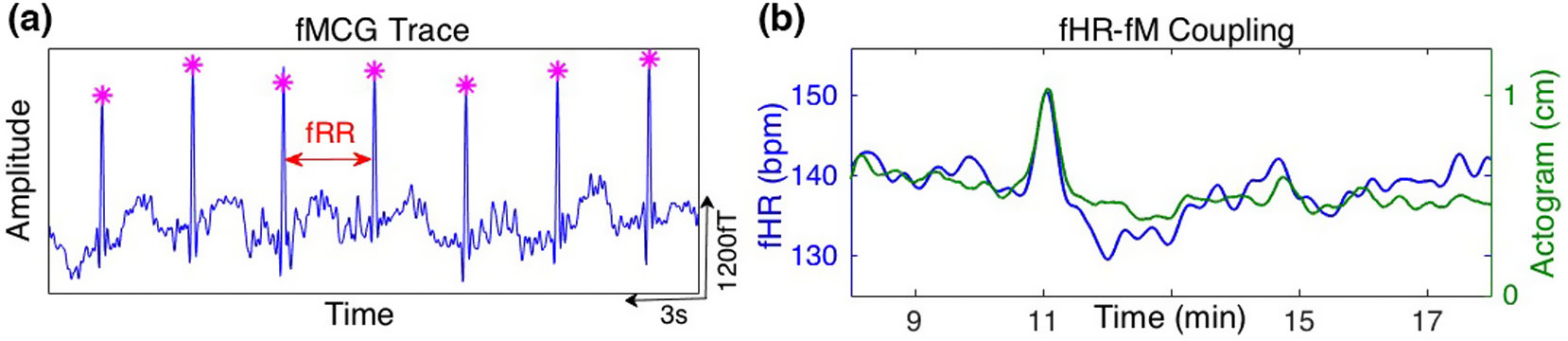
(a) fMCG trace on a single SARA sensor with the detected R markers (b) Coupling of fetal movement and heart rate acceleration.

**FIGURE 2:**
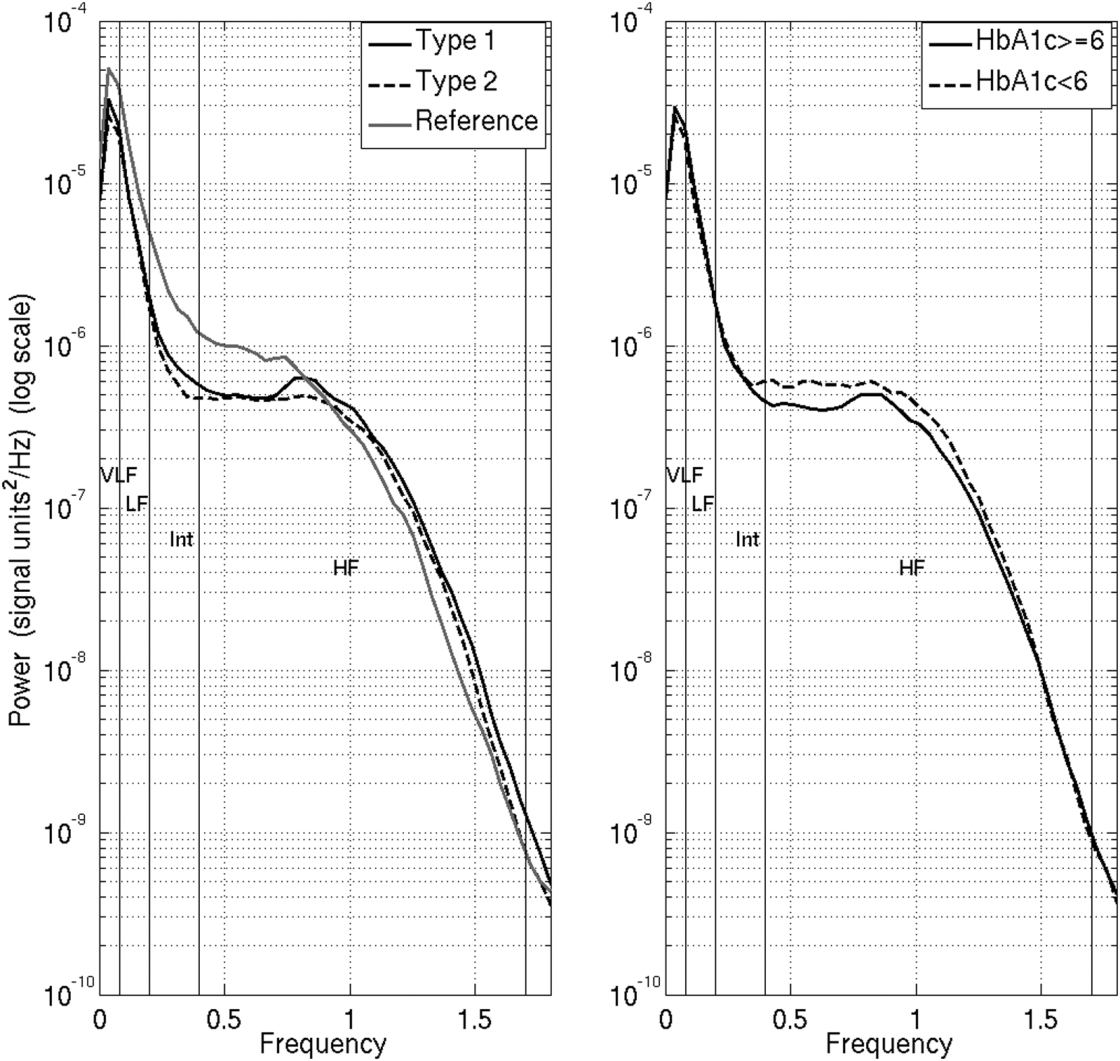
Spectral distribution in log scale for each of the groups by normative and diabetic type (left) and by glycemic control (right).

Figure 1a displays a representative fMCG trace that was plotted for a single SARA channel together with identified R markers for the first 3 s of the recording. Fetal R markers similar to the ones illustrated in the figure were used in the computation of fHRV metrics. Figure 1b displays a sample fHR‐fM coupling event that occurred around the 11th minute of the recording from the same Type 2 participant. Figure 2 shows the spectral distribution in log scale for each of the groups, both by normative and diabetic type and dichotomization by glycemic control.

### 3.1 Comparisons based on diabetic type

The maternal and fetal characteristics of our population are summarized in Table 1. According to the Kruskal–Wallis test, no significant difference among the Type 1, Type 2, and Reference groups was detected for maternal age, parity, smoking status, GA, fetal gender, birth weight, or APGAR scores at the 5th minute. In contrast, the three groups manifested statistically significant differences for pre‐pregnancy BMI, where the Type 2 and Type 1 groups had around 50% and 20% higher BMI, respectively, than the Reference group (*p* < 0.001). Maternal origin also differed notably between groups. Whites made up >90% of the Reference group, but represented only two thirds of Type 1 diabetics and only a quarter of Type 2 diabetics (*p* < 0.001). In addition, the Type 1 and Type 2 groups showed significant differences in the diabetes‐specific variables. Compared to their Type 2 counterparts, the Type 1 diabetics had elevated HbA_1c_ levels that were 36% elevated on the mmol/mol scale and 23% elevated on the % scale (*p* = 0.003). As expected, pre‐pregnancy insulin use was 100% among Type 1 diabetics, but only 42% among Type 2 diabetics (*p* = 0.014). Since one fetus from the Type 2 group was diagnosed with congenital heart disease, we excluded that participant from further analyses. Hence, we extracted fHRV and fHR‐fM coupling parameters for nine Type 1, 18 Type 2, and 12 Reference participants.

**TABLE 1:** Maternal and fetal characteristics of the groups based on diabetes type.

Maternal and fetal characteristics		Type 1 *N* = 9	Type 2 *N* = 19	Reference *N* = 12	*p* value
Maternal age (year)		24.9 (4.4)	28.9.0 (5.7)	27.9 (2.9)	0.10
Parity (count)		1.0 (1.2)	1.6 (1.3)	0.6 (0.5)	0.067
BMI (kg/m^2^)		29.1 (8.2)	36.1 (8.6)	24.5 (3.4)	**<0.001**
HbA_1c_	(mmol/mol)	60 (10.9)	44 (15.3)	NA	‐‐‐‐‐^a^ **0.003**
	(%)	7.6 (1.0)	6.2 (1.4)		
Birth weight (kg)		2.9 (1.3)^b^	3.1 (0.6)	3.5 (0.6)	0.17
APGAR score		8.4 (0.9)^b^	7.7 (2.1)	8.8 (0.8)^c^	0.63
GA (week)		32.8 (2.7)	34.2 (3.9)	33.5 (3.6)	0.62
Fetal gender	(F)	5 (56)	6 (32)	5 (42)	0.48
	(M)	4 (44)	13 (68)	7 (58)	**<0.001**
Maternal origin	(W)	6 (67)	5 (26)	11 (92)	
	(B)	3 (33)	12 (63)	1 (8)	
	(O)	0 (0)	2 (11)	0 (0)	
Smoker	(Y)	1 (11)	3 (16)	0 (0)	0.36
	(N)	8 (89)	16 (84)	12 (100)	
Insulin use before preg.	(Y)	9 (100)	8 (42)	NA	**0.014**
	(N)	0 (0)	11 (58)		

*Note*: Maternal origin: (W) White, (B) Black, (O) Other. Data for the continuous characteristics (first 7 variables in the top half of the table) are presented as mean (SD). Data for the binary and categorical characteristics (last 5 variables in the bottom half of the table) are presented as *n* (%). Statistically significant differences (*p* < 0.05) are in bold.

^a^ Not tested for HbA_1c_ in mmol/mol.

^b^ *N* = 8.

^c^ *N* = 5.

Table 2 contains the unadjusted group means and SDs of fHRV and fHR‐fM coupling parameters for each group, together with the GA‐adjusted group differences with 95% CIs obtained from the ANCOVA models. In the time domain, our results in Table 2 show that none of the parameters had a GA‐adjusted group difference with a 95% CI that was significant. This observation means that none of the fHRV parameters in the time domain had a statistically significant group difference after GA adjustment. However, in the frequency domain, some of the parameters had GA‐adjusted differences that achieved statistical significance. Specifically, when compared to the Reference group after adjusting for GA, the Type 2 diabetics were 50% lower on VLF (mean difference = −0.7 natural logs with 95% CI from −1.4 to −0.1 natural logs), 63% lower on LF (mean difference = −1 natural logs with 95% CI from −1.8 to −0.2 natural logs), and 50% lower on Total Power (mean difference = −0.7 natural logs with 95% CI from −1.3 to −0.1 natural logs). Further, Int frequency band also showed a similar behavior. When Type 1 diabetics were compared to the Reference group while adjusting for GA, they were 65% higher on the VLF/LF ratio (mean difference = 0.5 natural logs with 95% CI from 0.2 to 0.9 natural logs). Additionally, the fHR‐fM coupling parameters had two GA‐adjusted differences that were statistically significant. First, the fM rate increased 65% among Type 1 diabetics compared to Type 2 diabetics, with a log‐scale average difference (95% CI) of 0.5 (0.0, 1.0) natural‐log units (Table 2). Second, the coupling index decreased 63% among Type 1 diabetics compared to the reference group, with a log‐scale average difference (95% CI) of −1.0 (−2.0, −0.1) natural‐log units (Table 2).

**TABLE 2:** Fetal heart rate variability, fetal movement, and coupling parameters for the groups based on diabetes type.

	Type 1	Type 2	Reference	Difference (95% confidence interval)		
	*N* = 9 mean (SD)	*N* = 18^a^ mean (SD)	*N* = 12 mean (SD)	Type 1 vs. Reference	Type 2 vs. Reference	Type 2 vs. Type 1
Mean fHR (bpm)	147 (6)	143 (5)	143 (6)	4.2 (−0.9, 9.3)	0.4 (−4.0, 4.7)	−3.8 (−8.6, 1.0)
Mean fRR (ms)	410 (17)	422 (16)	422 (18)	−12 (−26.9, 3.0)	−1.7 (−14.4, 11.0)	10.2 (−3.9, 24.3)
SDNN (ms)	18.3 (6.5)	15.7 (4.8)	18.9 (7.2)	−0.4 (−5.8, 5.0)	−3.4 (−8.0, 1.2)	−3 (−8.1, 2.1)
RMSSD (ms)	6.02 (2.33)	6.15 (1.88)	6.83 (2.05)	−0.8 (−2.6, 1.1)	−0.8 (−2.3, 0.8)	0 (−1.7, 1.7)
ApEn	0.89 (0.46)	1.17 (0.34)	1.06 (0.45)	−0.2 (−0.5, 0.2)	0.1 (−0.2, 0.4)	0.3 (0.0, 0.6)
pNN20	1.38 (2.38)	1.18 (1.71)	1.75 (2.32)	−0.4 (−2.2, 1.5)	−0.6 (−2.2, 1.0)	−0.2 (−2.0, 1.5)
pNN15	3.74 (5.11)	3.66 (4.29)	5.22 (7.19)	−1.4 (−6.4, 3.6)	−1.7 (−5.9, 2.6)	−0.3 (−5, 4.4)
pNN10	6.54 (7.09)	6.89 (6.48)	9.14 (10.5)	−2.4 (−9.7, 4.8)	−2.5 (−8.7, 3.7)	0 (−6.9, 6.8)
Poincare‐SD1	4.26 (1.65)	4.34 (1.33)	4.82 (1.45)	−0.5 (−1.8, 0.8)	−0.5 (−1.6, 0.6)	0 (−1.2, 1.2)
Poincare‐SD2	25.47 (9.17)	21.81 (6.76)	26.17 (10.37)	−0.4 (−8.1, 7.3)	−4.8 (−11.3, 1.7)	−4.4 (−11.6, 2.9)
Log(VLF) (ms^2^/Hz)	−13.922 (0.686)	−14.289 (0.909)	−13.563 (0.923)	−0.4 (−1.1, 0.4)	**−0.7 (−1.4, −0.1)**	0.4 (−0.4, 1.1)
Log(LF) (ms^2^/Hz)	−15.285 (1.1)	−15.421 (1.099)	−14.407 (0.966)	−0.9 (−1.8, 0.1)	**−1 (−1.8, −0.2)**	0.2 (−0.7, 1.1)
Log(Int) (ms^2^/Hz)	−16.346 (1.062)	−16.437 (0.81)	−15.567 (1.247)	−0.8 (−1.7, 0.1)	**−0.9 (−1.6, −0.1)**	0.1 (−0.8, 0.9)
Log(HF) (ms^2^/Hz)	−15.068 (0.808)	−15.103 (0.582)	−14.719 (0.595)	−0.3 (−0.9, 0.2)	−0.4 (−0.9, 0.1)	0.1 (−0.5, 0.6)
Log(Total Power) (ms^2^/Hz)	−13.35 (0.681)	−13.562 (0.719)	−12.88 (0.836)	−0.5 (−1.1, 0.2)	**−0.7 (−1.3, −0.1)**	0.2 (−0.4, 0.9)
Log(VLF/LF)	1.36 (0.53)	1.13 (0.34)	0.84 (0.34)	**0.5 (0.2, 0.9)**	0.3 (0, 0.6)	0.2 (−0.1, 0.6)
Log(VLF/HF)	1.15 (0.82)	0.81 (0.97)	1.16 (0.73)	0 (−0.8, 0.8)	−0.3 (−1, 0.4)	0.3 (−0.4, 1)
Log(LF/HF)	−0.22 (0.87)	−0.32 (1.16)	0.31 (0.73)	−0.5 (−1.4, 0.4)	−0.6 (−1.4, 0.1)	0.1 (−0.8, 0.9)
fM (counts/minute)	0.666 (0.224)	0.412 (0.251)	0.461 (0.307)	0.4 (−0.1, 0.9)	−0.1 (−0.6, 0.3)	**−0.5 (−1.0, 0.0)**
Coupled fM (counts/minute)	0.065 (0.085)	0.064 (0.082)	0.103 (0.073)	−0.3 (−1.1, 0.5)	−0.6 (−1.3, 0.0)	−0.3 (−1.2, 0.5)
Coupling Index (%)	0.089 (0.106)	0.206 (0.298)	0.317 (0.212)	**−1.0 (−2.0, −0.1)**	−0.7 (−1.5, 0.1)	0.3 (−0.6, 1.3)
Coupling Latency (s)	1.89 (2.00)	2.82 (2.60)	3.5 (3.83)	−1.6 (−5.2, 1.9)	−0.5 (−3.7, 2.7)	1.1 (−2.9, 5.1)
Coupled fM Dur. (s)	10.16 (4.16)	12.11 (3.81)	11.38 (2.86)	−1.1 (−4.9, 2.6)	−0.4 (−3.8, 3.0)	0.7 (−3.5, 5.0)

*Note*: The “Mean (SD)” columns show group means (SDs) unadjusted for GA. The “Difference (95% Confidence Interval)” columns show GA‐adjusted differences with 95% confidence intervals (CIs) from ANCOVA models with Gestational Age (GA) as the continuous covariate. GA‐adjusted differences for count‐based coupling variables (fM, coupled fM, and coupling index) are adjusted differences in natural logs of count rates from negative‐binomial regressions with Group as the class variable and GA as the continuous covariate. Statistically significant differences (*p* < 0.05) are in bold.

^a^ One participant excluded—diagnosed with heart defect.

### 3.2 Comparisons based on glycemic control

Out of 27 participants included in the analyses, all nine Type 1 subjects and eight Type 2 subjects had HbA_1c_ values ≥6%. These 17 participants were categorized into the poor‐control group, while the other 10 Type 2 participants were assigned to the good‐control group. Table 3 shows maternal and fetal characteristics for each group. Similar to the previous comparison, in Table 4, neither the time domain parameters nor the fHR‐fM‐coupling parameters had a statistically significant GA‐adjusted group difference. Only one frequency domain parameter had a GA‐adjusted difference that achieved statistical significance. For the ratio between power bands VLF/LF, the poor‐control diabetics showed a 49% increase in this metric compared to the good‐control diabetics (mean difference (95% CI) = 0.4 (0.1, 0.7) natural logs; Table 4).

**TABLE 3:** Maternal and fetal characteristics of the groups based on HbA_1c_ levels.

Maternal and fetal characteristics		HbA_1c_ ≥ 6 *N* = 17	HbA_1c_ < 6 *N* = 11	Reference *N* = 12	*p* value
Maternal age (year)		27.6 (4.8)	27.7 (6.9)	27.9 (2.9)	0.98
Parity (count)		1.4 (1.5)	1.5 (1.0)	0.6 (0.5)	0.13
BMI (kg/m^2^)		32.0 (7.4)	36.6 (10.7)	24.5 (3.4)	**0.003**
HbA_1C_	(mmol/mol)	58 (13.1)	34 (5.5)	NA	‐‐‐‐‐^a^
	(%)	7.5 (1.2)	5.3 (0.5)		**<0.001**
Birth weight (kg)		3.0 (1.0)^b^	3.1 (0.6)	3.5 (0.6)	0.17
APGAR score		8.2 (1.2)^b^	7.5 (2.5)	8.8 (0.8)^c^	0.67
GA (week)		33.3 (3.4)	34.5 (4.0)	33.5 (3.6)	0.73
Fetal Gender	(F)	7 (41)	4 (36)	5 (42)	0.96
	(M)	10 (59)	7 (64)	7 (58)	
Maternal Origin	(W)	7 (41)	4 (36)	11 (92)	**0.001**
	(B)	10 (59)	5 (46)	1 (8)	
	(O)	0 (0)	2 (18)	0 (0)	
Smoker	(Y)	1 (6)	3 (27)	0 (0)	0.071
	(N)	16 (94)	8 (73)	12 (100)	
Insulin use before preg.	(Y)	14 (82)	3 (27)	NA	**0.014**
	(N)	3 (18)	8 (73)		
Diabetic type	T1D	9 (53)	0 (0)	NA	**0.007**
	T2D	8 (47)	11 (100)		

*Note*: Maternal Origin: (W) White, (B) Black, (O) Other. Data for the continuous characteristics (first 7 variables in the top half of the table) are presented as mean (SD). Data for the binary and categorical characteristics (last 5 variables in the bottom half of the table) are presented as *n* (%). Statistically significant differences (*p* < 0.05) are in bold.

^a^ Not tested for HbA_1c_ in mmol/mol.

^b^ *N* = 8.

^c^ *N* = 9.

**TABLE 4:** Fetal heart rate variability, fetal movement, and coupling parameters for the groups based on HbA_1c_ levels.

	HbA_1c_ ≥ 6 *N* = 17 mean (SD)	HbA_1c_ < 6 *N* = 10^a^ mean (SD)	Difference (95% confidence interval)
Mean fHR (bpm)	145 (6)	143 (6)	1.3 (−3.7, 6.4)
Mean fRR (ms)	416 (17)	420 (18)	−3.7 (−18.3, 10.9)
SDNN (ms)	16.5 (5.2)	16.7 (6.1)	0.3 (−4.3, 5.0)
RMSSD (ms)	6.0 (2.11)	6.3 (1.88)	−0.2 (−1.9, 1.5)
ApEn	1.06 (0.46)	1.10 (0.27)	−0.05 (−0.4, 0.3)
pNN20	1.27 (2.2)	1.21 (1.4)	0.04 (−1.6, 1.7)
pNN15	3.61 (4.93)	3.81 (3.86)	−0.2 (−4.2, 3.7)
pNN10	6.47 (6.92)	7.29 (6.20)	−0.8 (−6.6, 4.9)
Poincare‐SD1	4.24 (1.49)	4.44 (1.34)	−0.1 (−1.4, 1.1)
Poincare‐SD2	22.94 (7.38)	23.18 (8.57)	0.5 (−6.1, 7.0)
Log(VLF) (ms^2^/Hz)	−14.225 (0.695)	−14.069 (1.093)	−0.2 (−0.9, 0.6)
Log(LF) (ms^2^/Hz)	−15.6 (0.957)	−14.995 (1.218)	−0.6 (−1.5, 0.3)
Log(Int) (ms^2^/Hz)	−16.621 (0.925)	−16.043 (0.702)	−0.6 (−1.3, 0.2)
Log(HF) (ms^2^/Hz)	−15.116 (0.711)	−15.051 (0.566)	0 (−0.6, 0.5)
Log(Total Power) (ms^2^/Hz)	−13.595 (0.648)	−13.315 (0.784)	−0.3 (−0.9, 0.3)
Log(VLF/LF)	1.38 (0.41)	0.93 (0.23)	**0.4 (0.1, 0.7)**
Log(VLF/HF)	0.89 (0.81)	0.98 (1.14)	−0.1 (−0.9, 0.7)
Log(LF/HF)	−0.48 (0.91)	0.06 (1.23)	−0.6 (−1.4, 0.3)
fM (counts/minute)	0.528 (0.305)	0.443 (0.188)	0.2 (−0.3, 0.7)
Coupled fM (counts/minute)	0.046 (0.066)	0.095 (0.099)	−0.46 (−1.3, 0.4)
Coupling index (%)	0.127 (0.242)	0.235 (0.271)	−0.7 (−1.5, 0.2)
Coupling latency (s)	1.96 (2.13)	3.34 (2.71)	−1.9 (−4.8, 1.1)
Coupled fM Dur. (s)	10.57 (4.51)	12.79 (2.52)	−1.0 (−5.6, 3.6)

*Note*: The “Mean (SD)” columns show unadjusted group means (SDs) of untransformed data. The “Difference (95% Confidence Interval)” columns show GA‐adjusted differences with 95% confidence intervals (CIs) from ANCOVA models with Gestational Age (GA) as the continuous covariate. GA‐adjusted differences for count‐based coupling variables (fM, coupled fM, and coupling index) are adjusted differences in natural logs of count rates from negative‐binomial regressions with Group as the class variable and GA as the continuous covariate. Statistically significant differences (*p* < 0.05) are in bold.

^a^ One participant excluded—diagnosed with heart defect.

## 4 DISCUSSION

It is known that fHRV is regulated by the ANS, fetal sleep/wake cycles, and acidemia (Obstetricians, A.C.o. and Gynecologists, 2010; Schneider et al., 2018; van Laar et al., 2008, 2014; Van Leeuwen et al., 2013). fHRV provides an insight into regulation of the cardiovascular system by the ANS. While the fHRV time domain measures show the increased variability and complexity, the frequency parameters show that both sympathetic activity and vagal modulation increases with gestational age. Beyond 32 weeks, increased baroreceptor reflex and the presence of respiratory sinus arrhythmia reflect the maturation of the vagal activity (Schneider et al., 2008). In the frequency domain, the HF band is primarily associated with parasympathetic and respiratory activity while LF represents primarily sympathetic activity while not much importance is given to Int band due to low power content (Hamoud et al., 2019; Schneider et al., 2008). The LF/HF ratio is indicative of sympathovagal balance as the fetus matures and reflects ANS development. Further, Schneider et al. (Schneider et al., 2018) attributes VLF to baseline fluctuations while the VLF/LF and VLF/HF ratios describe the baseline fluctuations in relation to sympathovagal and vagal modulation, respectively. The fHR‐fM coupling coordinated with heart rate accelerations further show the advances in sympathetic activity. There is evidence that the maternal high‐risk conditions including diabetes and hypertension can alter this maturation process and this will be reflected in fHRV and fHR‐fM coupling parameters (Govindan et al., 2007).

In our study, we did not find any significant changes in fHRV time domain measures while a few frequency domain and fHR‐fM coupling measures showed significant differences in participants with maternal pregestational diabetes. Specifically, we found the fetuses of Type 1 diabetic mothers exhibited a higher VLF/LF ratio compared to fetuses of non‐diabetic mothers while none of the individual bands were significant. In the case of Type 2 diabetic mothers, the fetus exhibited lower VLF and LF compared to fetuses of non‐diabetic mothers while none of the ratios were significant. While categorizing in terms of glycemic control, mothers with poorly regulated HbA_1c_ exhibited lower VLF/LF ratios compared to fetuses of mothers with good glycemic control. Although there were significant differences in some of the fHRV frequency domain fHR‐fM coupling parameters in diabetic mothers, the effect of diabetes on fHRV and fetal ANS remained inconclusive based on the cohort we studied.

Although our observations did not show a strong evidence, a study by Fehlert et al. on fetuses of gestational diabetics (Fehlert et al., 2017) did observe difference in fHRV parameters when compared to normative pregnant mothers. In their study, the authors followed the similar protocol as published in Linder et al. (2014, 2015) and monitored short‐term changes in fetal heart activity. They found that there was a difference between the gestational‐diabetic and control groups in both time and frequency domain of fHRV measures with regard to SDNN, LF, and HF bands during the second hour of oral glucose tolerance test (Fehlert et al., 2017). Also, we did not observe any significant difference in time domain fHRV measures although there have been a few reported studies on fetuses of pregestational diabetic mothers where sonographic cardiac‐function changes and cardiac structural changes were observed in the first and third trimester, respectively, in comparison to the normative control group (Depla et al., 2021; Rizzo et al., 1991; Russell et al., 2008; Turan et al., 2011). Specific structural changes included a thickened interventricular septum and overall cardiac hypertrophy noted in the third trimester. These changes may be due to the decreased cardiac diastolic and global function observed in the first trimester (Russell et al., 2008). They proposed that maternal hyperglycemic states influence the developing heart's structure and function, and concluded that intrauterine acidemia and hypoxia, as induced by maternal hyperglycemia, contributed to the fetal autonomic dysfunction by altering the hypoxia inducible‐factor pathway (Russell et al., 2008, 2016).

Additionally, there are very limited comparative HRV studies on fetuses and neonates of pregestational diabetic mothers. Both the fetal (Hamoud et al., 2019) and neonatal (Russell et al., 2016) studies, are specifically on Type 1 diabetes and report alterations in sympathetic and parasympathetic balance reflected in the frequency domain related to the LF/HF ratio. The fetal study (Hamoud et al., 2019) on 26 Type 1 and healthy controls show a shift toward parasympathetic predominance as opposed to the report on 38 neonates born to Type 1 diabetic mothers where Russell et al. (Russell et al., 2016) observed a sympathetic predominance. We did not observe any significant LF/HF changes in either direction; although, our spectral analysis was based on electrophysiological recordings similar to Russell et al. (2016) on neonates as opposed to less precise fetal CTG recordings by Hamoud et al. (2019).

As mentioned earlier, our frequency bands were different than adults and were based on fetal electrophysiological studies on normative fetuses. In the study by David et al. (2007), it was observed that VLF has a sympathetic component, while LF has a parasympathetic component. They hypothesized that VLF/LF would reflect the sympathovagal balance. Further, they found a significant decrease in the VLF/LF and VLF/HF ratio across gestational age, but not in the LF/HF. We found there are significant differences in Type 1, where there was an increment in the VLF/LF ratio compared to the Reference group while a similar increase can be seen when comparing the groups based on glycemic control. The poor‐control group had a larger VLF/LF ratio than the good‐control group. However, for the Type 2 group, we observed around 40% less VLF and LF than the Reference group. Although we found isolated variations in VLF and LF and VLF/LF ratios, the fact that it is not consistently observed across all bands and their ratios including LF/HF, makes it difficult to conclusively comment on sympathovagal balance based on David et al.'s hypothesis (David et al., 2007). Due the exploratory nature of the study it is difficult to describe the mechanism beyond what we have hypothesized. We believe that the frequency domain HRV parameters as they relate to the sympathovagal relationship deserves to be further investigated in a larger cohort of fetuses in future studies.

It has been established that as gestational age advances, the coupling index increases while coupling latency decreases (DiPietro et al., 1996). Therefore, fHR‐fM coupling is thought to be related to fetal ANS maturation. The fetuses of the Type 1 group appear to have a statistically significant larger number of fM compared to the Type 2 group. Furthermore, the decreases in coupling index of 72% among Type 1 diabetics compared to non‐diabetics (which was statistically significant) and of 46% in the poor‐control group compared to the good‐control group (which was not statistically significant) may indicate there is slower maturation in these groups.

The strengths of this study include the non‐invasive assessment of fetal heart and ANS development in pregnancies affected by pregestational diabetes. To our knowledge, there have been no reports of detailed fetal heart variability analysis from direct electrophysiological recordings in participants with pregestational diabetes.

The main limitation of our study is the small sample size, especially for the Type 1 group. Also, our Reference group spanned the same GA range as the diabetics, but were not matched individually to them by GA or by other factors such as maternal origin that could potentially affect the data. Further, it has been reported that maternal origin can influence fHRV (Marie et al., 2015; Tagliaferri et al., 2017). Tagliaferri et al. (Tagliaferri et al., 2017) reported significant differences in HRV parameters including LF and HF while Marie et al. (Marie et al., 2015) observed lower short‐term variability in fetuses of Black pregnant women in comparison to White women. Our Reference group was predominantly White (92%) as compared to the other two groups especially in Type 2 and this imbalance could have influenced our comparative analysis.

As previously noted, we did not adjust for multiple comparisons, in order not to inflate Type II (i.e., false‐negative) error in this modestly powered exploratory study. Our decision not to do so is in keeping with recommendations by Rothman (1990) and Saville (1990). Saville in particular, recommends for general use of the unrestricted least significant difference procedure, with the understanding that this should be viewed as a hypothesis generator rather than a method for simultaneous generation and testing of hypotheses.

We plan to follow‐up on the hypotheses generated from this exploratory study by performing an adequately powered prospective study that can be controlled through concurrent enrollment of diabetic and non‐diabetic subjects that meet well‐defined inclusion/exclusion criteria. Concurrent‐enrollment designs without matching, generally achieve good GA balance on their own, but can be modified to include matching for maternal origin and/or other factors of interest. Further, we plan to include fetal behavioral‐state analysis including sleep‐ and awake‐state classification.

## 5 CONCLUSION

In summary, our study demonstrates the unique ability to determine the fetal ANS status using a non‐invasive biomagnetic technique starting as early as 28 weeks of GA in pregestational diabetic pregnancies. Further, there were some deviations in fHRV and fHR‐fM coupling parameters in the fetuses of pregestational diabetic mothers compared to non‐diabetic but the effect of fHRV related to fetal ANS and sympathovagal balance did not show strong evidence, potentially due to the limited sample size. There are reports of altered HRV and sympathovagal balance in neonates born to pregestational diabetic mothers, thus, we need further studies to investigate the origin of the effect occurring in the prenatal period.

## AUTHOR CONTRIBUTIONS

HE and SB designed the study. LM, DE, and HE analyzed data and JW, SB, KW, HP, and ES contributed to discussions. LM and ES performed the statistical analyses. LM, JW, and HE drafted the manuscript with contributions from DE, SB, KW, ES, and HP.

## FUNDING INFORMATION

This work was supported by the Sturgis Foundation for Diabetes Research, College of Medicine, Office of Research Intramural Grant Program, National Institutes of Health – NIBIB grant R01‐EB007826 and NICHD grant R01‐HD105412.

## CONFLICT OF INTEREST STATEMENT

None of the authors reported any financial interests or potential conflicts of interest other than disclosed grant funding. Dr. Whittington is an active duty member of the Armed Forces. The views expressed are those of the author(s) and do not reflect the official policy of the Department of the Navy, Department of Defense, or the US Government. She is a military service member. This work was prepared as part of her official duties. Title 17 U.S.C. 105 provides that “Copyright protection under this title is not available for any work of the United States government.” Title 17 U.S.C. 101 defines a United States government work as a work prepared by a military service member or employee of the United States government as part of that person's official duties.

## ETHICS STATEMENT

All procedures performed in studies involving human participants were in accordance with the ethical standards of the institutional and/or national research committee and with the 1964 Helsinki Declaration and its later amendments or comparable ethical standards. The study was approved by the UAMS Institutional Review Board (Protocol Numbers: #04234 on June 14th, 2017 and # 206700 on June 5th, 2017). All the participants provided informed written consent.
